# Confocal Laser Endomicroscopy in the Study of Colonic Mucosa in IBD Patients: A Review

**DOI:** 10.1155/2012/525098

**Published:** 2012-03-05

**Authors:** Francesca Salvatori, Saverio Siciliano, Francesco Maione, Dario Esposito, Stefania Masone, Marcello Persico, Giovanni D. De Palma

**Affiliations:** Department of General Surgery Geriatrics Oncology and Advanced Technology, Center of Excellence for Technical Innovation in Surgery (ITC), Section of Diagnostic and Therapeutic Endoscopy, School of Medicine and Surgery, University of Naples Federico II, 80131 Naples, Italy

## Abstract

Confocal laser endomicroscopy (CLE) is one of several novel methods that provide real-time, high-resolution imaging at a micronscale via endoscopes. CLE and related technologies are often termed “virtual biopsy” as they simulate the images seen in traditional histology. Recently, the use of CLE was reported in the study of colonic mucosa in patients with inflammatory bowel diseases and in particular in patients affected by ulcerative colitis. CLE has the potential to have an important role in management of IBD patients as it can be used to assess the grading of colitis and in detection of microscopic colitis in endoscopically silent segments. Moreover, CLE can be used in surveillance programs especially in high-risk patients. This report aims to evaluate the current data on the application of confocal endomicroscopy in clinical gastroenterology and particularly in the study of colonic mucosa in UC patients.

## 1. Introduction

Endoscopy has a recognized important role in diagnosis and management in inflammatory bowel disease (IBD). 

It can distinguish between Crohn's disease (CD) and ulcerative colitis (UC), assess activity, extension of the disease, and response to therapy, and it permits surveillance, especially in long-standing UC and extensive CD colitis patients, for cancer and dysplasia. 

Mucosal biopsy is a critical component of endoscopic examination for patients with suspected IBD to differentiate IBD from other causes of colitis such as bacterial infection, ischemia, and NSAID use; biopsy specimens can help differentiate CD from UC. Mucosal biopsy also helps to establish the extent of colon that is inflamed, which aids in determining prognosis, directing appropriate medical and surgical therapy and stratifying risk for dysplasia.

Moreover, histological findings have an important role in predicting relapse, because patients with acute inflammatory infiltrates seen on histological assessment are more likely to experience relapse than are those without infiltrates. Furthermore, some studies suggest that severity of inflammation is a risk factor for colorectal neoplasia in UC [1].

Colonoscopy underestimates the extent of disease compared with histology, and at present the extent of colitis (pancolitis, left-sided colitis, or proctitis) should be based on histologic examination rather than on endoscopy. Furthermore the assessment of inflammation activity by conventional colonoscopy is inaccurate in the prediction of acute inflammation in some cases, especially for those seeming to be in remission as evaluated by conventional colonoscopy.

Individuals with long-standing UC and extensive CD colitis are at increased risk for development of dysplasia and colorectal cancer (CRC) and should undergo colonoscopic surveillance. Surveillance of patients with ulcerative colitis consists of taking targeted and random biopsies. Biopsy specimens of the colon in patients with documented pancolitis should be obtained in all 4 quadrants every 10 cm from the cecum to the rectum, to obtain a minimum of 32 biopsy samples. Biopsy specimens should be obtained from strictures, mass lesions, and macroscopic abnormalities. The presence of high-grade dysplasia or multifocal low-grade dysplasia in flat mucosa and dysplasia-associated lesional mass (DALM) is an indication for colectomy.

Taking many biopsies is time consuming, carries a low but non negligible risk of secondary hemorrhage, and has only moderate sensitivity for neoplasia detection especially when random biopsies are taken. 

In recent years many efforts have been done to improve the diagnostic power of endoscopy, and technology has provided the endoscopist new advanced tools such as chromoendoscopy, high-resolution and magnification endoscopy, narrow-band imaging and autofluorescence.

These new technologies offer enhanced endoscopic images that can predict the histopathological diagnosis of the examined mucosa and target the biopsy to suspected and representative spot of the mucosa. Reported results have shown that these procedures had a better relation with histology than did conventional colonoscopy. However, some impractical aspects of dye-based chromoendoscopy, such as longer procedure times and different dye stainings and washing techniques, contributed to its limited application. Although these factors do not affect the “virtual” chromoendoscopy methods, such as narrow-band imaging (NBI) or Fujinon intelligent color enhancement (FICE), an extensive review by the ASGE on these methods shows modest and variable accuracy [2].

Confocal laser endomicroscopy (CLE) is a newly introduced technique which provides real-time high-magnified images of the gastrointestinal mucosa during endoscopic examination. It offers the chance to the endoscopist to have in vivo visualization of the histology of the mucosal epithelium with its cellular and subcellular structures. CLE during endoscopy has shown high agreement with the real histology of the tissue. The current potential indications for CLE imaging are broad and include almost all the cases in which endoscopic biopsy is needed [1, 3–5]. 

To date various studies have addressed the potential of CLE in UC patients evidencing that this technique can have an important role in assessing the extension and the activity of disease and in targeting biopsies, reducing the number of useless biopsies and improving the early detection of dysplasia.

In this paper, we will focus on the role of CLE as applied to UC patients with a particular emphasis on the potential of CLE in in surveillance programs.

## 2. Confocal Systems/Methods

CLE can be performed currently with 2 devices: one integrated into an endoscope (Pentax, Tokyo, Japan, herein termed eCLE) and one as a stand-alone probe (herein termed pCLE) capable of passage through the accessory channel of most endoscopes (Cellvizio, Mauna Kea Technologies, Paris, France).

The Pentax confocal endoscopes (Pentax EG-3870CIK upper endoscope and EC3870CILK colonoscope) generate simultaneously endoscopic and confocal images, so the endoscope working channel is available to use and can capture images at different depth levels from 0 to 250 *μ*m.

The Cellvizio endomicroscopy system probe can be used with any endoscope through the working channel and has different probes which enable different scanning depth levels (40–70 *μ*m, 70–130 *μ*m-55–65 *μ*m). Single video frames are reconstructed by a special computer algorithm (mosaicing) in an image with an enlarged field of view. The video mosaicing technique as applied is based on a hierarchical framework algorithm that is able to recover a globally consistent alignment of the input frames, to compensate for motion-induced distortions. The resulting video mosaics combine all moving images, cancel motion artifacts, and reconstitute panoramas of the tissues.

There are no data, at present, comparing pCLE with eCLE to demonstrate the superiority of any single system. pCLE has several advantages and disadvantages compared with eCLE. Advantages include the greater versatility of pCLE probes, which can be used in conjunction with virtually any endoscope (high-resolution endoscopes, NBI, cholangioscope, etc.), ad hoc usage (such as when a lesion is detected with a normal endoscope), and acquisition at video frame rate of 12 frames/s, allowing in vivo imaging of capillary flow. Disadvantages include a slightly lower resolution (approximately 1 *μ*m compared with 0.7 *μ*m for eCLE) and smaller field of view (240–600 *μ*m).

Unequivocally, this technology is best used in conjunction with other “red-flag” techniques because of its minute scanning area and thus is only appropriate for classification of tissue at a site already detected by standard or optically enhanced endoscopy. Ideally, the “red-flag” techniques such as chromoendoscopy, narrow-band imaging, or autofluorescence imaging should be used to screen the mucosa for “areas of interest,” which can then be interrogated by CEM for a “histological” diagnosis. The best combination in UC surveillance is between chromoendoscopy and CLE as chromoendoscopy is the gold standard to detect regions of suspicion that can be examined by CLE to confirm intraepithelial neoplasia and guide immediate therapy.

A fluorescent contrast agent is needed to achieve high-contrast images using CLE. Potentially suitable agents in humans are fluorescein, acriflavine, tetracycline, or cresyl violet. The most commonly used in studies have been fluorescein and acriflavine. Topical acriflavine is highly specific for labeling acidic constituents staining cellular nuclei of superficial layers of the mucosa and may allow better differentiation between intraepithelial neoplasia and cancer of the GI tract. However, because of the risk of mutagenesis related to this agent, its use in humans has been reduced.

Sodium fluorescein is the agent of choice as it is nonmutagenic and relatively inexpensive and it has been safely used for decades in ophthalmology. 

It is highly safe with most common side effects being short-term yellowish skin discoloration and bright-yellow-colored urine. Transient and minor nausea and vomiting were reported during angiography. Serious side effects, such as anaphylaxis or cardiac or respiratory effects, are extremely rare, and to date, have not been recorded in CLE.

Intravenous injection of 1.0–5.0 mL of a 10% solution enables visualization of individual cells with strong contrast of the capillary network. Cell nuclei and mucin are not stained by fluorescein and therefore appear dark. 

Fluorescein, after binding serum albumin and staining the vascular space, diffuses in the extravascular space and stains the epithelium and the stromal tissue allowing visualization of enterocytes, cellular infiltrate, surface epithelial cells, blood vessels, and red blood cells.

## 3. Confocal Images Evaluation/Classification

Kiesslich et al. in 2004 were the first who defined criteria for classification of e-CLE patterns of normal, regenerative, and neoplastic tissue based on evaluation of crypts and vascular architecture, named the Mainz confocal endomicroscopy criteria [7]. The Miami classification system was developed for p-CLE images, on the base of a consensus of p-CLE users reached during a meeting held in Miami, Florida, in February 2009 [8]. Due to the significant technical differences compared with e-CLE (smaller field of view, fixed depth), p-CLE images are not comparable to e-CLE images.

At present there is not a worldwide accepted classification of CLE images in UC, and this is certainly a limit of this technique. This reflects the fact that this is a recently introduced technology, and few centers have published studies on this technique up to now. The most used classifications are showed in Tables 1, 2, and 3 and are based on crypt architecture assessment and microvascular assessment [6, 9, 10].

**Figure 1:**
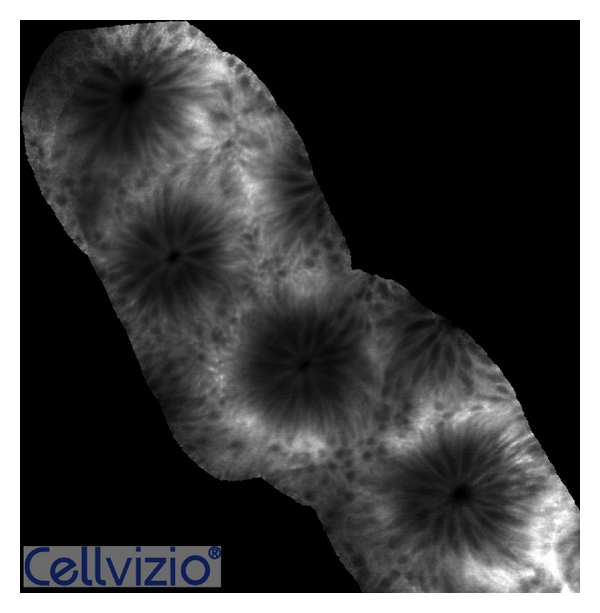
p-CLE fluorescein sodium 10% imaging of the normal colon showing hexagonal, honeycomb appearance with a regular-ordered network of capillaries demarcating the luminal crypt orifice. Surface crypt architecture was classically represented by ordered and regular crypt orifices covered by a homogeneous epithelial layer with visible “black-hole” goblet cells within the subcellular matrix.

**Figure 2:**
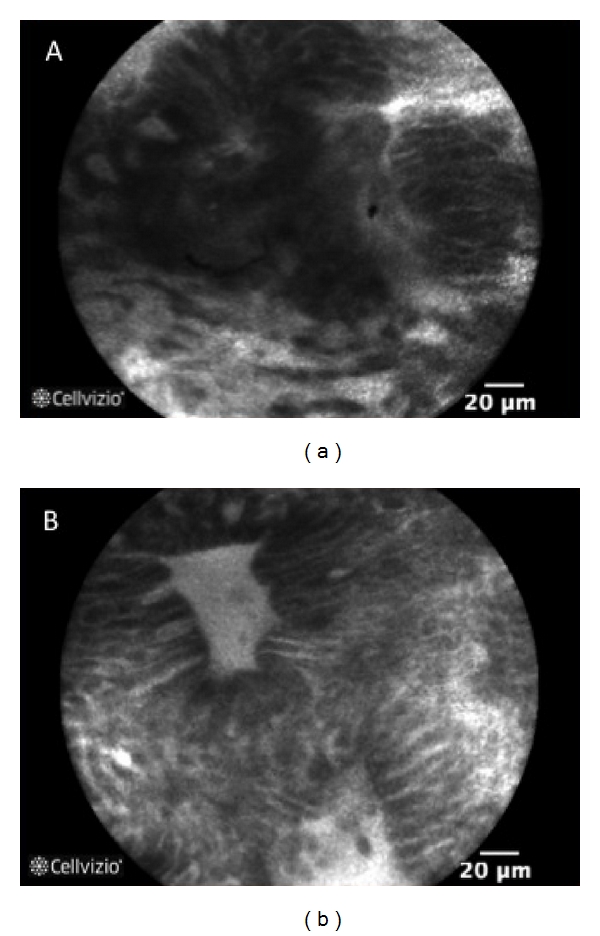
p-CLE fluorescein sodium 10% imaging examples of crypt types of patients in active ulcerative colitis. (a) crypt fusion and distortion; (b) dilation of crypt openings, with fluorescein leaks into the crypt lumen therefore making the lumen brighter than the surrounding epithelium.

The most frequent alterations in crypt architecture are represented by dilation of crypt openings, more irregular arrangement of crypts, enlarged spaces between crypt, crypt destruction and/or crypt fusion, and crypt abscess with fluorescein leaks into the crypt lumen (therefore making the lumen brighter than the surrounding epithelium) (Figures 1 and 2). Microvascular alterations are mainly represented by dilated, prominent branching vessels.

**Figure 3:**
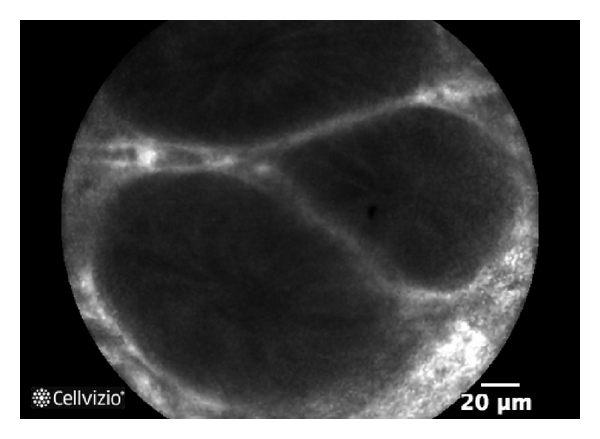
p-CLE fluorescein sodium 10% imaging of vessel architecture in ulcerative colitis: preserved hexagonal, honeycomb appearance with slightly dilated capillaries.

**Figure 4:**
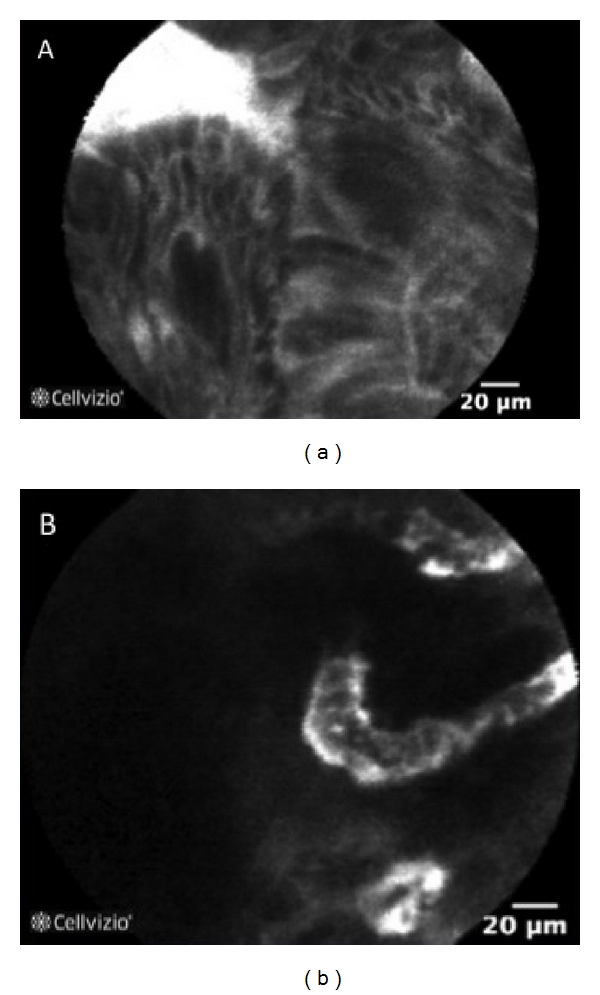
p-CLE fluorescein sodium 10% imaging of dysplastic epithelium in ulcerative colitis. (a) the architectural pattern of crypt is irregular, with epithelial thickness, villiform structures, and “dark” epithelial border. (b) the vessel architecture shows tortuous and dilated capillaries.

Dysplasia is characterized by “dark” cells, with mucin depletion and goblet cell/crypt density attenuation; the architectural pattern is irregular, as well as the epithelial thickness, with villiform structures and “dark” epithelial border. The blood vessels are dilated and irregularly branched, with poor orientation to adjunct tissue and fluorescein extravasation (Figures 3 and 4).

## 4. Clinical Application and Review of the Literature

CLE has the potential to have an important role in management of IBD patients. It cannot distinguish between CD and UC as it cannot be used to make a diagnosis, but it can assess the grading of colitis and detect microscopic colitis in endoscopically silent segments. Moreover, CLE can be used in surveillance programs especially in high-risk patients.

At present most of the literature is about the use of CLE in UC patients, to monitor disease activity and for surveillance.

Watanabe et al. [9] and Li et al. [6] reported on real-time inflammation activity assessment by CLE. The inflammation activity assessment includes crypt architecture, cellular infiltration, and vessel architecture. These studies evidenced that images taken with the CLE provided information that was equivalent to conventional histology, differentiating between active and nonactive CUC patients during ongoing endoscopy.

CLE may be useful particularly in the surveillance of patients with UC, where suspicious lesions can be evaluated in vivo, reducing the need for random biopsies by combining CLE with chromoendoscopy. 

Several randomized studies have shown that targeting biopsies with chromoendoscopy significantly increases dysplasia detection rates in patients with long-standing ulcerative colitis [11, 12]. Chromoendoscopy was demonstrated, in fact, to have a higher sensitivity than conventional white light colonoscopy in the detection of dysplasia in UC patients while it has a low specificity. CLE has a high specificity so it would be ideal to join the two techniques in cancer and dysplasia surveillance. 

In a randomized study on 161 patients with long-term UC, pan-chromoendoscopy has been used to detect flat or suspected lesion and targeted CLE of the detected lesions to differentiate between neoplastic and nonneoplastic tissue. By using this diagnostic approach, Kiesslich et al. detected 4.75 times more dysplastic lesions than with conventional endoscopy. There was a reduction in the number of biopsy specimens by half. In addition, CLE was able to predict neoplastic changes with an accuracy of 97.8% [13].

A pilot study on 22 patients of van den Broek investigated the feasibility and diagnostic accuracy of CLE in conjunction with NBI high definition endoscopy in surveillance of patients with long-standing UC [10]. 

They reported a diagnostic accuracy of 81%, with a moderate interobserver agreement, reflecting their minimal experience with this technique, that certainly needs a learning curve to obtain the best results. They also reported an additional time to colonoscopy of 30–40 minutes to capture images that were evaluated in a second time, and video image quality was than rated as good–excellent only in 69%. For sure, the time needed to perform CLE and to use it to have real-time evaluation of colonic mucosa and taking on-table decision is a limit to the general application of CLE in clinical practice.

Hurlstone et al. [2] assessed the clinical applicability and predictive power of the CLE for the in vivo differentiation of ALM and DALM in CUC. The in vivo diagnosis of DALM and ALM using CLE matched the histological evaluation, with a kappa coefficient of 0.91 and an accuracy of 97%. The study evidenced that ALM and DALM can be differentiated with a high overall accuracy, enabling the safe selection of patients suitable for endoluminal resection versus immediate referral for surgery. In a recent case report of our group, pCLE has been used to characterize a DALM in a long-standing UC with high correlation between CLE and standard histopathological examination [14].

## 5. Conclusions

CLE is a new technique that promises to be an important imaging tool in the management of patients with UC; it can be used to assess and score IBD activity and to monitor response to therapy and it has the potential to allow the developing of new activity markers, without the need for histological confirmation. 

It can avoid unuseful biopsy, precisely target biopsy on suspected area, and allow on-table management decision in surveillance.

This technology is best used in conjunction with other “red-flag” techniques and thus can be used for classification of tissue at a site already detected by standard or enhanced endoscopy. Ideally a red-flag technique such as chromoendoscopy should be used to screen the mucosa for areas of interest, to examine with CLE for a histological diagnosis.

The joining of the two techniques could lead to a higher diagnostic accuracy in surveillance endoscopy in UC patients and to the detection of neoplasia at an earlier and curative stage.

There are some limitations to the application in general practice: the need for a learning curve, the cost of the equipment the need for an extra time to enhanced colonoscopy, and the promising results in the literature being derived from still a few experienced centers. New multicenter studies are needed to assess the cost effectiveness of this technique for surveillance endoscopy in UC.

CLE is a new technology, with the potential, with appropriate training and careful patient selection, to become an important imaging modality in the complex clinical scenario of UC cancer and dysplasia surveillance.

## Figures and Tables

**Table 1:** Classification of crypt architecture by e-CLE assessment in ulcerative colitis [6].

CLE crypt architecture	Description
(A) normal	Regular arrangement and size of crypts
(B) chronic inflammation	Irregular arrangement of crypts, enlarged spaces between crypts
(C) acute inflammation	Dilation of crypt openings, more irregular arrangement of crypts, and enlarged spaces between crypts as compared to type B
(D) acute inflammation	Crypt destruction and/or crypt abscess

**Table 2:** Microvascular architecture by e-CLE assessment in ulcerative colitis [7].

Vessel architecture	Description
Normal	Hexagonal, honeycomb appearance that presents a network of capillaries outlining the stroma surrounding the luminal openings of the crypts
Inflammation-regenerative	Preserved hexagonal, honeycomb appearance with a slight increase in the number of capillaries
Dysplastic	Dilated and distorted vessels with increased leakage; irregular architecture, with little or no orientation to the adjoining tissue

**Table 3:** Assessment of crypt architecture and vessel architecture by p-CLE in ulcerative colitis [8].

Crypt architecture	Crypt fusion and distortion Bright epithelium
Vessel architecture	Dilated, prominent branching vessels
